# Hypomethylation of *HOXA4* promoter is common in Silver-Russell syndrome and growth restriction and associates with stature in healthy children

**DOI:** 10.1038/s41598-017-16070-5

**Published:** 2017-11-16

**Authors:** Mari Muurinen, Katariina Hannula-Jouppi, Lovisa E. Reinius, Cilla Söderhäll, Simon Kebede Merid, Anna Bergström, Erik Melén, Göran Pershagen, Marita Lipsanen-Nyman, Dario Greco, Juha Kere

**Affiliations:** 1 Folkhälsan Institute of Genetics, Helsinki, and Research Programs Unit, Molecular Neurology, University of Helsinki, Helsinki, Finland; 2 Department of Dermatology, Skin and Allergy Hospital, University of Helsinki and Helsinki University Hospital, Helsinki, Finland; 3 Department of Biosciences and Nutrition, Karolinska Institutet, Huddinge, Sweden; 4 Department of Women’s and Children’s Health, Karolinska Institutet, Stockholm, Sweden; 5 Institute of Environmental Medicine, Karolinska Institutet, Stockholm, Sweden; 6 Sachs’ Children’s Hospital, Södersjukhuset, Stockholm, Sweden; 7 Centre for Occupational and Environmental Medicine, Stockholm County Council, Stockholm, Sweden; 8 Children’s Hospital, University of Helsinki and Helsinki University Hospital, Helsinki, Finland; 9 Institute of Biotechnology, University of Helsinki, Helsinki, Finland; 10 Faculty of Medicine and Life Sciences & Institute of Biosciences and Medical Technology, University of Tampere, Tampere, Finland; 11 School of Basic and Medical Biosciences, King’s College London, Guy’s Hospital, London, UK

## Abstract

Silver-Russell syndrome (SRS) is a growth retardation syndrome in which loss of methylation on chromosome 11p15 (11p15 LOM) and maternal uniparental disomy for chromosome 7 [UPD(7)mat] explain 20–60% and 10% of the syndrome, respectively. To search for a molecular cause for the remaining SRS cases, and to find a possible common epigenetic change, we studied DNA methylation pattern of more than 450 000 CpG sites in 44 SRS patients. Common to all three SRS subgroups, we found a hypomethylated region at the promoter region of *HOXA4* in 55% of the patients. We then tested 39 patients with severe growth restriction of unknown etiology, and found hypomethylation of *HOXA4* in 44% of the patients. Finally, we found that methylation at multiple CpG sites in the *HOXA4* promoter region was associated with height in a cohort of 227 healthy children, suggesting that *HOXA4* may play a role in regulating human growth by epigenetic mechanisms.

## Introduction

Silver-Russell syndrome is an imprinting disorder characterized by severe pre-and postnatal growth retardation and typical clinical features, including relative macrocephaly, protruding forehead, body asymmetry and feeding difficulties^1^. Imprinting disorders affect the function of genes that are normally expressed predominantly or solely from one parental chromosome. Chromosomal abnormalities or changes in DNA methylation level can alter imprinted gene dosage and lead to phenotypes affecting growth or neurological functioning^2^. Opposite changes in the same imprinted genomic regions can lead to opposite growth phenotypes, such as growth restriction in SRS and overgrowth in Beckwith-Wiedemann syndrome (BWS), caused by hypo- and hypermethylation, respectively, of the imprinted H19-IGF2 region in 11p15 (H19/IGF2:IG-DMR) in a proportion of the cases^3^. SRS is a heterogeneous syndrome both in phenotype and in molecular etiology, but severe growth retardation is a central feature shared by the patients.

SRS patients can be divided into three subgroups based on their molecular etiology: maternal uniparental disomy of chromosome 7 (UPD(7)mat), loss of methylation on chromosome 11p15, (11p15 LOM; also known as hypomethylation of *H19*/*IGF2:*IG-DMR or H19 hypomethylation), and clinical SRS without any of these molecular findings. Approximately 10% of SRS patients have UPD(7)mat and 20–60% of SRS patients have 11p15 LOM^4–10^. Duplications and deletions at 11p15 affect <1% of SRS patients and even other molecular changes, such as UPD of chromosome 11 have been detected in single cases^3^. The remaining cases can be designated clinical SRS, which make up 30–70% of the SRS patients.

As all SRS patients share the same clinical diagnosis, while a substantial amount of patients test negative for molecular diagnosis, we hypothesized that there might occur further epigenetic changes that all subgroups of SRS share in common. To search for such genes or regions, we analyzed 44 SRS patients for methylation changes by the genome-wide Illumina Infinium HumanMethylation450K BeadChip assay that provides quantitative methylation data at more than 450 000 single CpG sites. In addition, we evaluated associations between the found methylation changes and severe growth restriction of unknown etiology (SGR), as well as growth in healthy children.

## Results

### Genome-wide methylation analysis pinpointed *HOXA4* hypomethylation as a common epigenetic change among SRS subgroups

**Figure 1:**
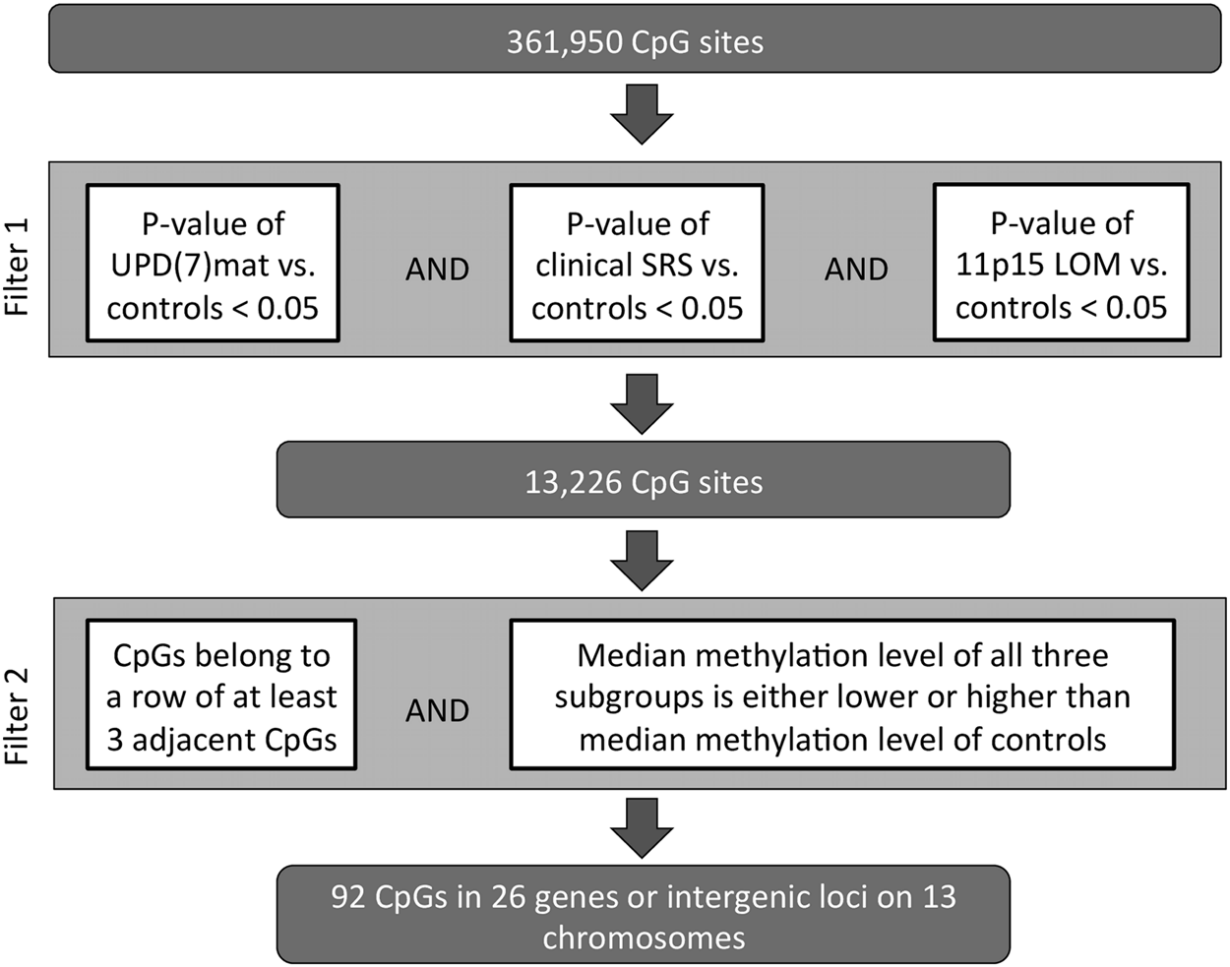
Filtering for common differentially methylated regions in SRS patients.

We used the Infinium HumanMethylation450K BeadChip (Illumina) to identify shared methylation changes in the three different SRS subgroups. We used a filtering approach (Fig. 1) to search for areas that were significantly differentially methylated in all subgroups compared to controls over a stretch of consecutive CpGs. In the first stage of filtering (filter 1), altogether 13,226 CpGs were found, in which the differential methylation between each of the three SRS groups compared to controls reached the empirical Bayes significance (nominal p-value < 0.05). We then chose regions that had at least three consecutive significant CpGs for all subgroups and discarded CpGs in which the methylation level of different groups varied to opposite directions in comparison to the controls (filter 2). This filtering process substantially narrowed down the potential areas and resulted in 92 CpGs located in 26 genes or intergenic loci on 13 different chromosomes (Supplementary Table 1). Of these CpGs, 66 showed only small differences in median Beta-value (<0.02) between each of the subgroups and controls. Altogether 16 CpGs showed median Beta-value differences >0.05 (5% absolute methylation level) for all three subgroups. Two of the 16 CpGs were located in an intergenic region on 5p15.33 and 14 were located in the *HOXA4* gene region on 7p15.2. *HOXA4* was the only area that showed at least 5% median methylation difference for all subgroups vs. controls for at least three consecutive significant CpGs. In total, *HOXA4* showed 12 consecutive probes with such differences. The differences for the individual groups in these 12 CpGs were also larger than with any other CpGs, ranging from 6.8–25% in UPD(7)mat, 7.1–21% in clinical SRS and 5.0–11% in 11p15 LOM patients. As a result of the filtering process of genome-wide methylation data, *HOXA4* emerged as a specific candidate for further study.

### *HOXA4* methylation levels differ among subgroups and are lowest near the *HOXA4* transcription start site

**Figure 2:**
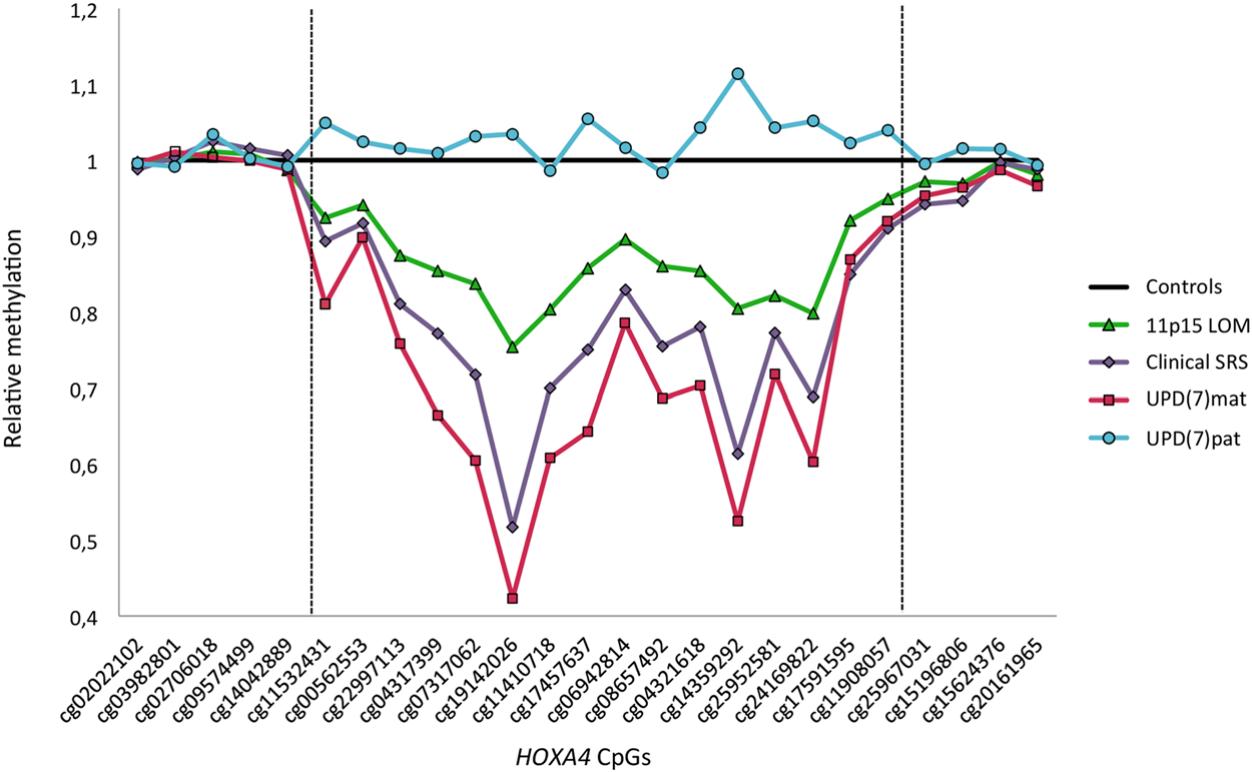
*HOXA4*. Median methylation levels of SRS subgroups relative to controls (1). Boundaries of the region in which *HOXA4* CpGs are hypomethylated in SRS groups relative to controls are marked with two dashed vertical lines.

**Figure 3:**
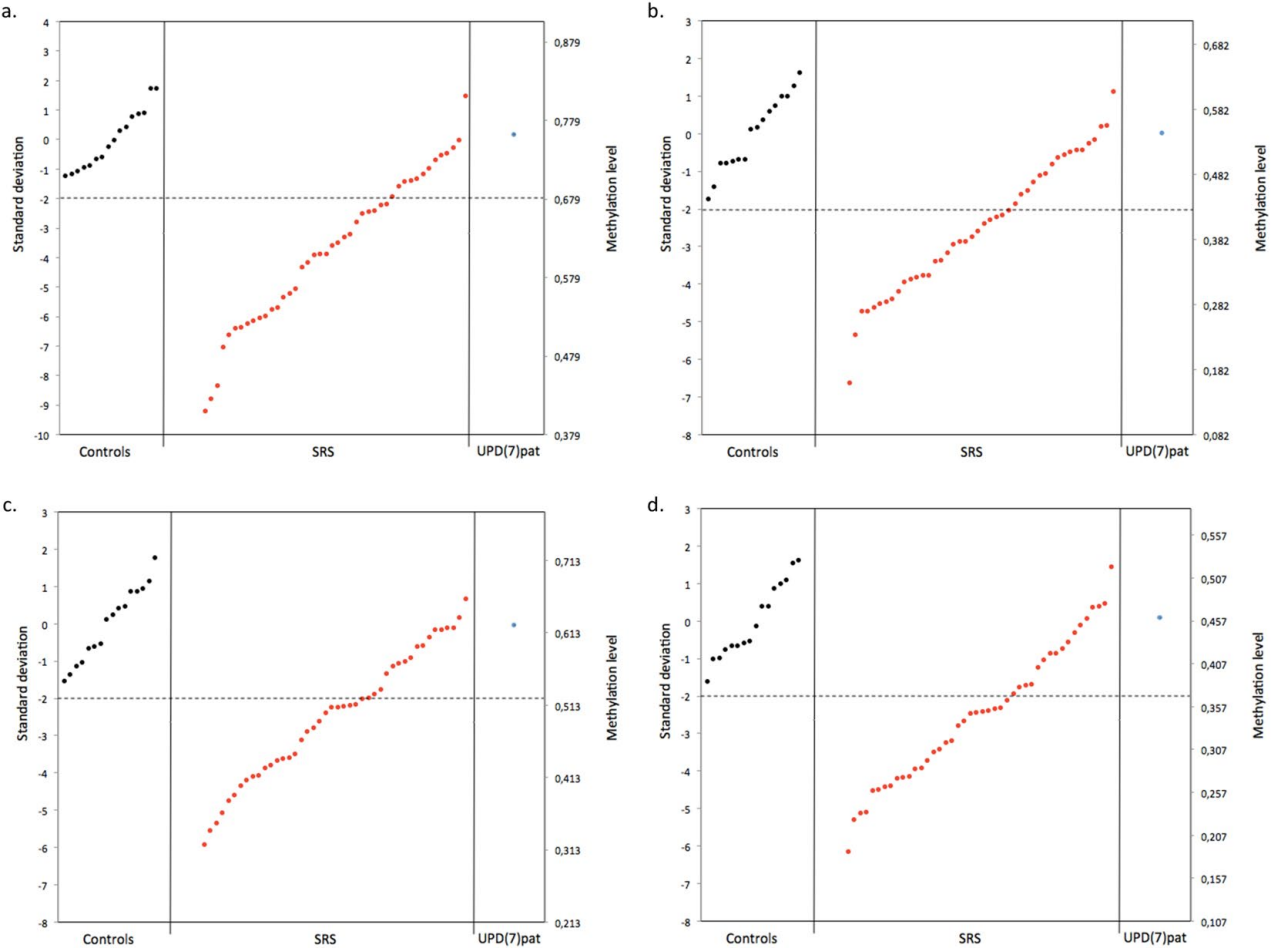
Methylation level at *HOXA4* cg22997113 (**a**), cg11410718 (**b**), cg08657492 (**c**) and cg04321618 (**d**). Individuals within each group are represented in ascending order of methylation. Dashed line represents -2 standard deviations of the arithmetic mean of control group methylation level.

All three SRS subgroups showed hypomethylation throughout the *HOXA4* region identified by filtering when comparing median methylation levels relative to controls (Fig. 2). This differentially methylated area included two stretches of consecutive CpGs: from cg11532431 to cg14359292 (chr7:27169674–27170892 in GRCh37/hg19 assembly), and from cg24169822 to cg11908057 (chr7:27170994–27171154). Localized between these stretches, cg25952581 (at chr7:27170961) did not reach the empirical Bayes significance for differential methylation between controls and 11p15 LOM group (p = 0.082), but clinical SRS and UPD(7)mat had significant methylation differences (p = 0.013 and p < 0.001 respectively), and the methylation pattern was similar to the pattern of other CpGs in the region. P-values for differential methylation between subgroups and controls for all *HOXA4* CpGs included in our filtering are shown in Table 1. For the majority of the CpGs, the median methylation level was lowest in the UPD(7)mat group, followed by clinical SRS, 11p15 LOM, and controls in ascending order. The methylation level of the single UPD(7)pat sample was higher than controls for most of the CpGs. Figure 3 shows examples of methylation levels at four individual CpG sites, where methylation level of most SRS patients fell below -2 SD and UPD(7)pat remained within the range of the control group’s methylation level. Some SRS patients showed extremely low hypomethylation patterns and were hypomethylated even as low as 8 SD below the mean of the control group at cg22997113.

**Table 1:** P-values for differential methylation for SRS subgroups (11p15 LOM, clinical SRS, UPD(7)mat) compared to controls for all *HOXA4* CpGs that passed quality control and were included in the filtering process of Illumina 450 K BeadChip assay data. Regulatory feature group as indicated by Illumina is shown in column “Regulatory”: “PROMOTER” = Promoter_Associated, “CELL TYPE” = Unclassified_Cell_type_specific. Relation to UCSC CpG island chr7:27169572-27170638 is shown in column “CGI”.

**Coordinates in hg19 (chr 7)**	**CpG ID**	**Regulatory**	**CGI**	**11p15 LOM**	**clinical SRS**	**UPD(7)mat**
27168609	cg02022102		N_Shore	0,1337	0,3379	**0,0345**
27168688	cg03982801		N_Shore	0,4355	0,2119	0,8381
27168780	cg02706018		N_Shore	0,9538	0,3056	0,5738
27168962	cg09574499		N_Shore	0,8489	0,2095	0,4154
27169208	cg14042889		N_Shore	0,6536	0,8850	0,6749
27169674	cg11532431	CELL TYPE	Island	**0,0011**	**0,0013**	**8,2E-06**
27169740	cg00562553		Island	**0,0052**	**0,0037**	**0,0002**
27170241	cg22997113	PROMOTER	Island	**0,0006**	**0,0001**	**1,5E-06**
27170313	cg04317399	PROMOTER	Island	**0,0309**	**0,0030**	**4,1E-05**
27170388	cg07317062	PROMOTER	Island	**0,0073**	**0,0014**	**8,4E-06**
27170394	cg19142026	PROMOTER	Island	**0,0061**	**0,0005**	**7,3E-06**
27170412	cg11410718	PROMOTER	Island	**0,0037**	**0,0004**	**3,3E-06**
27170717	cg17457637	PROMOTER	S_Shore	**0,0349**	**0,0011**	**1,4E-05**
27170819	cg06942814	PROMOTER	S_Shore	**0,0116**	**0,0004**	**1,9E-05**
27170832	cg08657492	PROMOTER	S_Shore	**0,0063**	**0,0001**	**4,4E-06**
27170880	cg04321618	PROMOTER	S_Shore	**0,0172**	**0,0009**	**9,7E-06**
27170892	cg14359292	PROMOTER	S_Shore	**0,0125**	**0,0001**	**9,8E-07**
27170961	cg25952581	PROMOTER	S_Shore	0,0818	**0,0125**	**0,0002**
27170994	cg24169822	PROMOTER	S_Shore	**0,0120**	**0,0005**	**5,4E-06**
27171051	cg17591595	PROMOTER	S_Shore	**0,0371**	**0,0006**	**0,0033**
27171154	cg11908057		S_Shore	**0,0108**	**0,0001**	**0,0031**
27171203	cg25967031		S_Shore	0,0804	**0,0035**	**0,0167**
27171213	cg15196806		S_Shore	0,0526	**0,0173**	0,0674
27171391	cg15624376		S_Shore	0,8751	0,8147	0,2402
27171401	cg20161965		S_Shore	0,0634	0,2399	**0,0240**

The identified differentially methylated area of *HOXA4* was located in the promoter region and co-localized with the CpG island at chr7:27169573–27170638. Approximately half of the differentially methylated CpGs were located within the CpG island and the rest within the proximal CpG island shore. According to SwitchGear Genomics transcription start sites, the *HOXA4* TSS is at chr7:27170399 and according to FANTOM5, at chr7:27170364–27170377 in the hg19 build (GRCh37/hg19 assembly). Cg19142026 at position chr7:27170394, the CpG with lowest relative median methylation of the *HOXA4* differentially methylated area for all SRS subgroups, was within 5-17 bp, respectively, of the TSS.

### *HOXA4* hypomethylation frequency was highest for UPD(7)mat, followed by clinical SRS and 11p15 LOM patients

**Figure 4:**
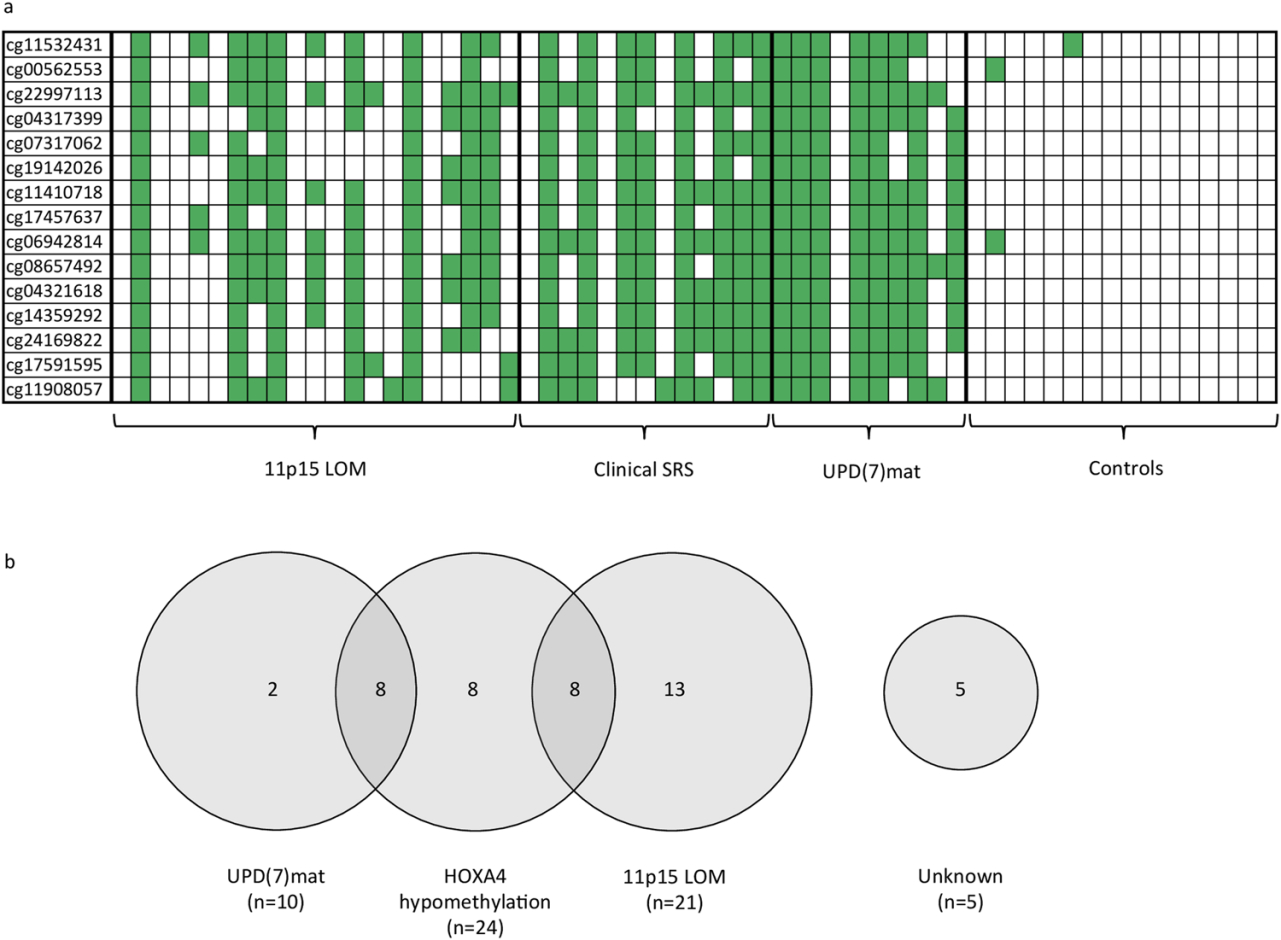
(**a**) *HOXA4* CpG methylation status represented by individuals in each SRS group. Each column represents one patient. Green color indicates methylation level below -2SD of the controls. (**b**) Molecular changes of SRS patients, n = 44. HOXA4 hypomethylation is present in 55% of the patients.

While group results showed the trend that UPD(7)mat patients had the lowest median level of methylation for CpGs in the *HOXA4* differentially methylated area, followed by the clinical SRS and 11p15 LOM groups, the methylation level varied between individuals within each subgroup. Figure 4(a) shows hypomethylation of individual SRS patients for CpGs in the *HOXA4* differentially methylated area. The frequency of *HOXA4* hypomethylation was highest for UPD(7)mat patients at 80%, followed by clinical SRS at 62% and 11p15 LOM group at 38%, when hypomethylation for an individual patient was defined by at least two-thirds of the CpGs being hypomethylated (<−2 SD). In addition to the patients that were hypomethylated in majority of the CpGs in the *HOXA4* area, three 11p15 LOM patients and two clinical SRS patients had hypomethylation in at least one-third of the *HOXA4* CpGs, demonstrating a partial deviation from normal methylation status in the area.

### Shared molecular etiology for SRS patients

Analysis of the methylation data of *HOXA4* region studied by the Illumina assay revealed that altogether 24 (55%) of the SRS patients were hypomethylated in at least two-thirds of the CpG sites at *HOXA4* differentially methylated area. Often both UPD(7)mat and *HOXA4* hypomethylation, or *HOXA4* hypomethylation and 11p15 LOM occurred in the same individual, as seen in Fig. 4(b). Either 11p15 LOM, UPD(7)mat, *HOXA4* hypomethylation or a combination of two changes was found in 39 out of 44 SRS patients (89%). Only 5 patients (11%) were left without a molecular change defined by the chosen criteria. In two of these patients, we observed partial hypomethylation in the *HOXA4* differentially methylated area.

### Frequency of 11p15 LOM was higher than previously tested

After initial analysis of our 44 SRS patients, we observed that more individuals had 11p15 LOM than had previously been identified. 15 of our 21 11p15 LOM SRS patients had been previously found hypomethylated with a restriction site-specific methylation method^11^. Of the six patients that had previously not been found hypomethylated, two had not been tested and four had been tested but found negative. Supplementary Table 2 summarizes our result, indicating that 21 of our patients had clear methylation changes in this area with at least two-thirds of the 36 CpGs hypomethylated (below -2 SD of the controls) in the region.

### EpiTYPER methylation assay revealed *HOXA4* hypomethylation in patients with severe growth restriction of unknown etiology (SGR)

**Figure 5:**
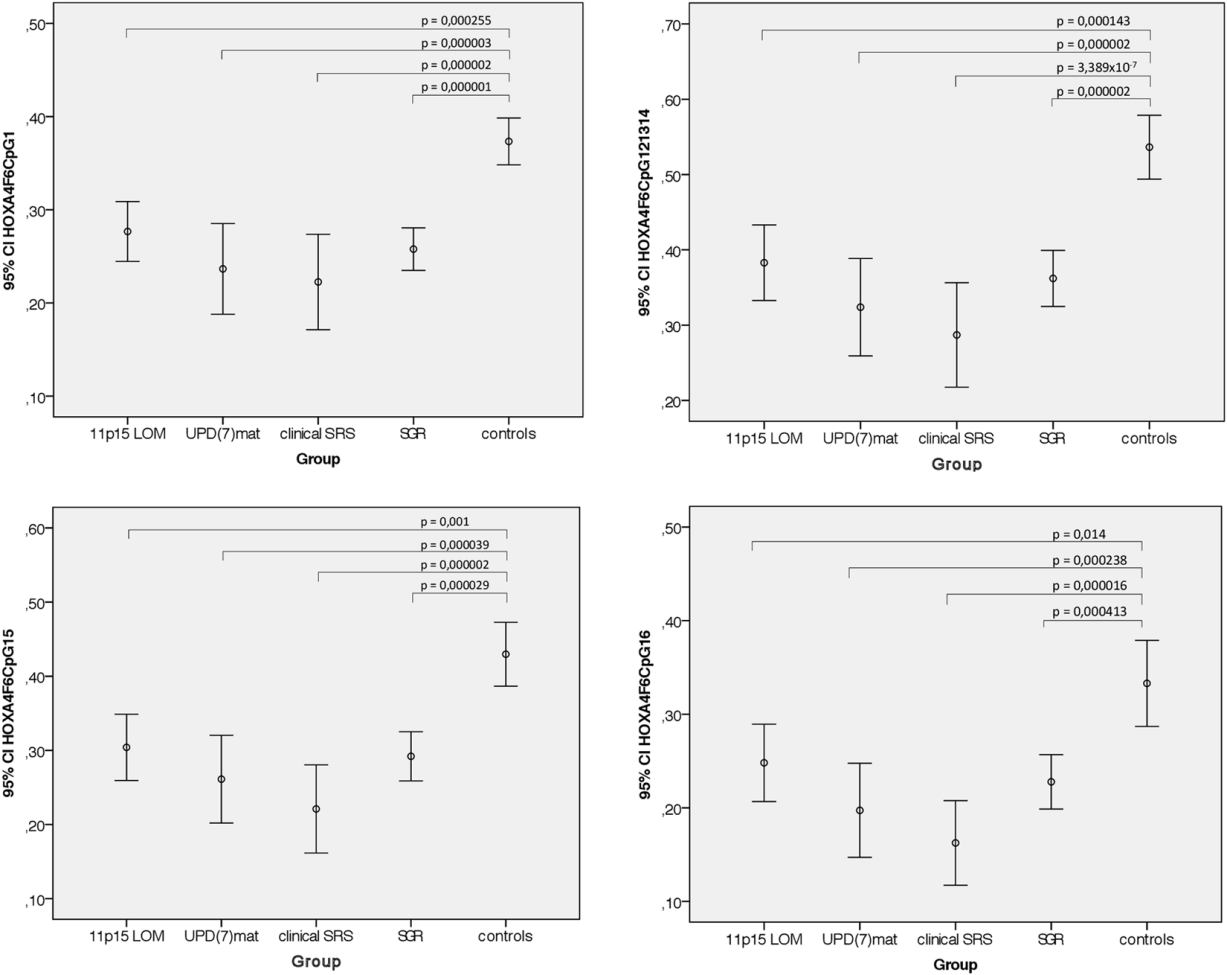
Methylation levels measured at four EpiTYPER sites for 11p15 LOM (n = 21), UPD(7)mat (n = 10), clinical SRS (n = 13), and SGR (n = 38, for HOXA4_F6_CpG_12.13.14 n = 37) patients compared to controls (n = 15). Comparisons made by 2-sided Dunnett t-tests.

We used the Sequenom EpiTYPER method to assay the methylation level of the *HOXA4* promoter region in order to validate results from the Illumina genome-wide methylation study and to study a group of children with SGR. The participants included 44 SRS patients, 16 controls and 39 children with SGR. The EpiTYPER assay spanned chr7:27170191–27170313, overlapping the area with significant methylation differences in the genome-wide methylation study. One CpG, cg04317399 at chr7:27170313, was covered by both Illumina 450 K and EpiTYPER, here named HOXA_F6_CpG_16. Analysis showed a strong correlation between the two methods, R^2^ = 0.86. (Supplementary Figure 1). Methylation levels of four different CpG sites (HOXA4F6CpG1, HOXA4F6CpG12.13.14, HOXA4F6CpG15, HOXA4F6CpG16) were measured for all participants. Statistically significant differences between the groups were determined by one-way ANOVA (HOXA4F6CpG1: F(4,92) = 10.831, p < 0.001; HOXA4F6CpG12.13.14: F(4, 91) = 11.549, p < 0.001; HOXA4F6CpG15: F(4,92) = 9.268, p < 0.001; HOXA4F6CpG16: F(4, 92) = 7.556, p < 0.001). Post hoc comparisons using 2-sided Dunnett t-tests indicated that the mean methylation levels of 11p15 LOM, UPD(7)mat, clinical SRS and SGR groups were statistically significantly different from the mean of the controls at all four studied sites (Fig. 5). The largest mean difference (I-J = −0.249, p = 3.389 × 10^−7^) was observed between clinical SRS (I) and controls (J) at HOXA4F6CpG12.13.14. Also, an analysis of individual SGR patients showed that 44% (17/39) of the patients were hypomethylated for at least two-thirds of the CpGs in the *HOXA4* promoter region at chr7: 27170191–27170313. Supplementary Table 3 shows individual methylation status of all SRS and SGR patients studied with the EpiTYPER assay. Thus, *HOXA4* hypomethylation was not limited to SRS patients, but also implicated in patients with SGR.

### Methylation level of *HOXA4* cg11908057 correlates with height in healthy children

**Figure 6:**
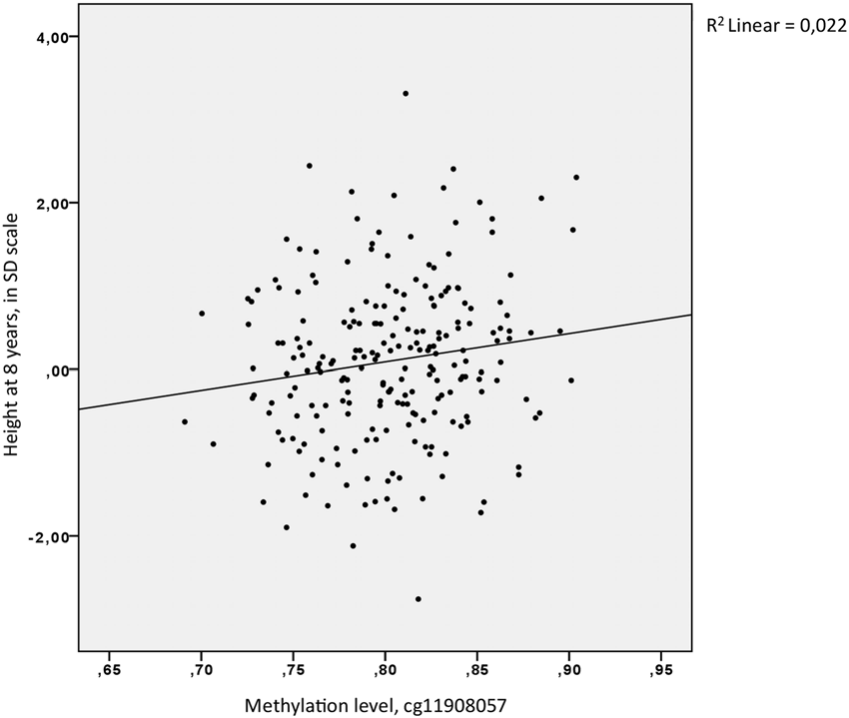
Height measurements (in SD scale) of BAMSE individuals (n = 225) at age 8 plotted against meathylation level of cg11908057 measured by Illumina 450 K BeadChip assay.

We studied correlation of methylation levels of *HOXA4* and height of altogether 227 healthy children from the BAMSE (Swedish abbreviation for Children, Allergy, Milieu, Stockhom, Epidemiology) cohort, a birth cohort of children born between 1994 and 1996 in Stockholm, Sweden. Methylation levels were measured with Infinium HumanMethylation450K BeadChip (Illumina)^12^. Sixteen CpGs of the *HOXA4* differentially methylated area were studied. We observed a statistically significant correlation between the methylation level of one of the *HOXA4* differentially methylated CpGs, cg11908057, and height at four years of age (r = 0.142, n = 227, p = 0.033), eight years of age (r = 0.148, n = 225 p = 0.026) and sixteen years of age (r = 0.164, n = 200, p = 0.020) by computing Pearson correlation coefficient. As methylation data for some of the CpGs did not meet criteria for normal distribution, additional analysis using all CpGs by computing Spearman correlations revealed statistically significant positive correlation between methylation level and height at sixteen years of age for 5 additional CpGs: cg04317399 (r = 0.146, n = 200, p = 0.040), cg19142026 (r = 0.139, n = 200, p = 0.050), cg04321618 (r = 0.149, n = 200, p = 0.036), cg14359292 (r = 0.140, n = 200, p = 0.047) and cg25952581 (r = 0.145, n = 200, p = 0.040). Cg11908057 demonstrated similar results as above: positive correlation was observed between methylation level and height at eight years of age (r = 0.140, n = 225, p = 0.036) and sixteen years of age (r = 0.147, n = 200, p = 0.038). Figure 6 demonstrates that the methylation level of cg11908057 in healthy children at eight years of age ranges from 0.69 to 0.91. This variation in methylation corresponds to a difference of 0.75 SD (or 4.5 cm) in height. In the same individuals, we studied correlation of height at age eight with some of the most strongly height-associated SNPs^13,14^ (Supplementary Table 4). In the tested SNPs, there was no significant association between height and genotype (Supplementary Figure 2). The results indicate that in our dataset of healthy-school aged children from the BAMSE cohort, the methylation level of cg11908057 at *HOXA4* was better in explaining height variation at age eight than some of the most strongly height-associated SNPs. We also used regulomeDB^15^ to study whether cg11908057 is localized in a potential regulatory protein-binding site. There was some evidence of binding of an enhancer of zeste homolog 2 (EZH2), a methyltransferase enzyme encoded by the *EZH2* gene on 7q36.1. Mutations of *EZH2* cause Weaver syndrome^16^, a pre- and postnatal overgrowth syndrome resulting in tall adult stature.

### Methylation level of *HOXA4* promoter region correlates with expression level of *HOXA4* in blood

**Figure 7:**
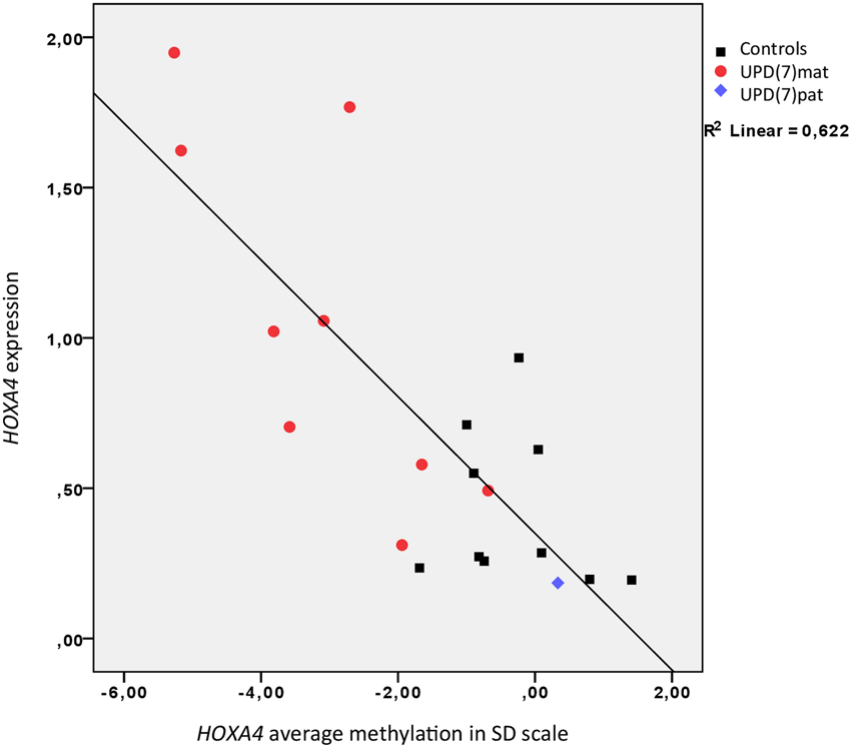
*HOXA4* expression level in blood measured by qPCR vs. average methylation level of *HOXA4* CpGs in SD scale. Average methylation was calculated from all *HOXA4* CpGs that showed significant differential methylation between all SRS groups and controls with Illumina 450 K analysis.

We used data previously published in Hannula-Jouppi *et al*.^17^, to study the correlation of *HOXA4* promoter region CpG methylation level and expression level of *HOXA4* in blood in nine UPD(7)mat patients, one individual with UPD(7)pat and ten controls. The average methylation level of the *HOXA4* region in SD scale correlated negatively with the expression level of *HOXA4* in blood, as demonstrated in Fig. 7. In all differentially methylated CpGs of *HOXA4*, we observed a statistically significant negative correlation between expression and methylation level by Spearman correlation analysis. The negative correlation was strongest for cg22997113 (r = −0.81, n = 20, p = 1.40 × 10^−5^). Data for other CpGs is listed in Supplementary Table 5.

## Discussion

We conducted a genome-wide methylation study, determining the methylation status of more than 450,000 CpG sites for UPD(7)mat, 11p15 LOM and clinical SRS patients, and aimed the data analysis at finding common methylation changes among these three groups. Initial analysis showed such changes in 26 genes or intergenic loci, and further analysis pinpointed *HOXA4* promoter hypomethylation as a major defect in SRS. *HOXA4* hypomethylation occurred in 55% of SRS patients, and was thus more common in our cohort than 11p15 LOM (48%) or UPD(7)mat (23%), and specifically, was found in eight 11p15 LOM (38%), eight UPD(7)mat (80%), and eight patients without any prior molecular diagnosis (62%). Furthermore, targeted methylation analysis revealed that *HOXA4* hypomethylation was also present in children with severe growth-restriction of unknown etiology. Further analysis of the *HOXA4* region identified multiple CpG sites where lower methylation level was associated with lower height in healthy school-aged children.

*HOXA4* belongs to the cluster of *HOXA* genes located on 7p15.2. The *HOXA4* hypomethylated region was located at chr7:27169674–27171154, with most CpGs classified as promoter-associated, and half of the CpGs located within the overlapping CpG island and the rest in the proximal island shore. CpG islands in somatic cells are usually unmethylated, and methylated promoter CpG islands are involved in long-term repression of, for example, imprinted genes^18^. Methylated CGIs are often observed near developmentally important genes, such as the *HOX* genes, and it is thought that the large numbers of potential CGI promoters within *HOX* loci may contribute to their regulation^19^. Relative *HOXA4* methylation level was lowest for all the SRS subgroups at cg19142026, in close proximity to the functionally relevant transcription start site. Lower methylation level is often related to transcriptional activation of genes, while hypermethylation can lead to transcriptional repression^16,20^. We observed that hypomethylation of the *HOXA4* promoter region was correlated with increased expression of *HOXA4* in blood, when using expression data from UPD(7)mat, UPD(7)pat and controls from our previously published study^17^. Other reports have shown that hypermethylation of *HOXA4* promoter region promotes inactivation of gene expression^21,22^. Altered methylation levels of the *HOXA4* promoter therefore seem to have functional relevance by affecting gene expression.

Several genes within the *HOXA* gene cluster (*HOXA2, HOXA3, HOXA4, HOXA5, HOXA6* and *HOXA11)* are predicted as maternally expressed imprinted genes^23^. In our previous study, *HOXA4* was monoallelically expressed and the expression of *HOXA4* was increased in whole blood of the UPD(7)mat group relative to controls^17^, supporting the prediction of *HOXA4* as a maternally expressed imprinted gene. In UPD(7)mat patients, *HOXA4* hypomethylation can therefore be explained by the occurrence of two maternal copies of chromosome 7, although the hypomethylation was not observed in all of the UPD(7)mat patients in our study. In 11p15 LOM patients, *HOXA4* hypomethylation could potentially be due to multi-locus imprinting disturbance (MLID). MLID is reported to be present in 15–38% of 11p15 LOM patients^1^.

The *HOX/Hox* genes in human and other species are highly interesting in the context of growth disorders. They encode a family of transcription factors that regulate early developmental morphogenetic processes and are important regulators of anterior-posterior axis development^24^. Ten of the 39 *HOX* genes have been associated with human disorders^25^, such as *HOXA13* with Hand-foot-genital syndrome (HFGS). Human phenotypes of the loss-of-function mutations in genes of the *HOXA* cluster include facial dysmorphisms, limb anomalies, cardiac defects and urogenital malformations. As the *Hox* genes are highly conserved, human phenotypes are very similar to their mouse orthologs. *Hox* gene knockouts in mouse affect the skeleton, primary vertebral column and slightly less frequently the bones of the forelimb and hindlimb. Mouse *Hoxa4* loss-of-function homozygote phenotype showed skeletal abnormalities including partial anterior transformation of C3, posterior transformation of C7 to T1, presence of a C7 cervical rib and malformation of the sternum^26,27^. The reported *Hox* gene defects that often affect bone morphology suggest that disturbance in the normal pattern of *HOXA4* methylation may also have relevance considering skeletal malformations in SRS patients. Interestingly, in 3-M syndrome, a growth disorder with a phenotype very similar to SRS, downregulation of *IGF2* and upregulation of several *HOX* genes has been reported^28^.

Our study also included two rare samples, a UPD(7)pat and a UPD(7q31-qter)mat, in order to enable further investigation of the potential findings^29^. The methylation level of the UPD(7)pat sample was higher than the median methylation level of controls in most differentially methylated CpGs of *HOXA4*, but still within normal range (+/−2SD). It is interesting to note that there is no growth abnormality in this individual or other reported UPD(7)pat cases^29–31^. The segmental UPD(7q31-qter)mat was hypomethylated at 10 of the *HOXA4* CpGs, even though *HOXA4* (at 7p15.2) is located outside of the segmental UPD(7q31-qter)mat region. Further studies are required to better understand this observation.

Unexpectedly, we found that some of the patients that had been tested negative for 11p15 LOM previously, were actually positive according to our results from the genome-wide methylation study. Prickett *et al*. reported similar findings, where methylation-sensitive RFLP PCR method failed to detect 11p15 LOM patients while 11p15 LOM was found by the Infinium Human Methylation 450 K BeadChip array^32^. These findings suggest that the genome-wide methylation assay may be more sensitive at detecting hypomethylated patients than some other methods.

Methylation levels may vary in different tissues and time in development. Our results are limited to methylation levels present at the time of sampling in accessible whole blood tissue. Variation in the methylation level of the different white blood cell populations in whole blood can complicate interpretation of whole blood methylation profiles^33^. In the *HOXA4* region, such variation between blood cell types was not observed, and therefore potential differences in blood cell populations of the participants should not affect our results. Also genome-wide methylation studies of human blood have reported age-related modifications in specific CpGs during early childhood^34^, adulthood^35^ and from newborn to elderly in a meta-analysis^36^. Our patients included both children and adults, while control samples were all adults. CpGs in the *HOXA4* region were, however, not among the reported age-modified loci, and therefore age of the participants is not expected to significantly affect our results.

Our genome-wide study of SRS patients found hypomethylation at multiple adjacent CpGs in the promoter region of the developmentally important *HOXA4* gene. Our results indicated that *HOXA4* hypomethylation was not specific to SRS, but was also present in children with severe growth restriction of unknown etiology. Our study in healthy children indicated an effect in the same direction, showing the association of hypomethylation of multiple *HOXA4* CpGs with short stature. It is also worth mentioning that in *HOXA3*, a gene adjacent to *HOXA4*, an association between birth weight and methylation has been observed in two CpGs^37^, demonstrating that the region may be important in regulating body size. We hypothesize that *HOXA4* plays a role in growth-regulating pathways affecting SRS, related growth disorders, and also stature in general. Remarkably, the effect size of *HOXA4* methylation in our healthy child cohort was larger than found for any of nine highly significantly height-associated SNPs. As growth disorders share many diagnostic features, it is plausible that underlying genetic or epigenetic defects are shared across closely related phenotypes. Our results suggest a new candidate region potentially relevant in the pathogenesis of SRS, other growth disorders and growth in general.

## Methods

### Patients and controls

We studied 44 SRS patients. The patients included three subgroups based on molecular findings: 21 patients with 11p15 LOM, 10 patients with UPD(7)mat, and 13 patients negative for both 11p15 LOM and UPD(7)mat, assigned as clinical SRS. One paternal uniparental disomy 7 (UPD(7)pat)^27^ sample without growth retardation was also included for comparison. One of the patients in UPD(7)mat had segmental UPD(7q31-qter)mat. SRS diagnoses were made based on criteria for SRS diagnosis in use at the time of evaluation. Information of birth weight, birth length, postnatal growth, body asymmetry, protruding forehead, relative macrocephaly and feeding difficulties are listed in Supplementary Table 6. Birth length and weight for gestational age in SD scale are according to Finnish growth references^38^. Ten control blood samples were obtained from adult volunteers of normal height. Six additional adult control samples were from healthy blood donors^31^. 39 patients with severe growth restriction of unknown etiology (SGR) were included in a targeted analysis of *HOXA4* hypomethylation. SGR patients had severe growth restriction but did not fulfill criteria for SRS diagnosis at the time of evaluation. Information on birth length and birth weight for gestational age, and postnatal growth is listed in Supplementary Table 7. The majority of the SGR patients were born small for gestational age (SGA) and all of them had postnatal growth restriction. Methylation data from the BAMSE study^12^ were used to test for correlation with height in healthy children. The study was approved by the Ethical Review Board of the Hospital for Children and Adolescents, Helsinki University Central Hospital, Helsinki, Finland (SRS and SGR patients and controls) and the Ethical Review Board North at Karolinska Institutet (BAMSE, blood donor controls). Informed consent was obtained from all study participants. Clinical investigations have been conducted according to the Declaration of Helsinki.

### Genome-wide methylation analysis with Illumina 450K BeadChip assay

Methylation analysis was carried out on 44 SRS patients, one individual with UPD(7)pat, and 10 control samples. DNA from EDTA blood samples was extracted using the FlexiGene DNA Kit (Qiagen) according to manufacturer’s instructions. 500 ng of DNA was bisulfite converted with the EZ-96 Methylation Kit (Zymo research corporation) according to the manufacturer’s instructions. Samples from 27 patients, UPD(7)pat and 10 control samples were analyzed at the Karolinska Institutet Bioinformatic and Expression Analysis (BEA) Core Facility, and additional 17 samples at the Mutation Analysis Core Facility (MAF, www.maf.ki.se). Bisulfite-treated DNA was amplified, fragmented and hybridized to the HumanMethylation450 BeadChip (Illumina) according to standard Illumina protocol and imaging was performed using Illumina iScan scanner. Additional data from similarly performed methylation analysis of six whole blood control samples^31^ produced at MAF were integrated into the analysis. Data produced at different facilities were comparable (data not shown). Methylation data for the BAMSE cohort had also been produced at MAF.

### Bioinformatics analysis of the Illumina 450K BeadChip assay for SRS patients and controls

The.idat files were imported in R and analyzed using the minfi package^39^. After quality check, the data were normalized using the subset-quantile within array normalization (SWAN) method^40^. The probes overlapping with known SNPs as well as probes prone to cross-hybridization problems were removed, rendering 361,948 CpG sites for further analysis. The M-values were extracted and the batch effects removed using the ComBat method^41^. Differentially methylated CpG sites were obtained using linear models (y~subgroup + gender) and pairwise comparisons with empirical Bayes as implemented in the limma package^42^. The differentially methylated CpG sites with p-value < 0.05 after Benjamini and Hochberg correction for multiple testing were considered for further analysis.

### Filtering process for common methylation differences in genome-wide methylation data

We analyzed 361,948 CpGs for significant differential methylation of the UPD(7)mat, 11p15 LOM, and clinical SRS groups in comparison to controls. At the first stage of filtering (filter 1), we selected CpGs that showed significant differential methylation with a nominal p-value < 0.05 (empirical Bayes significance) for each of the three patient groups when compared to controls. Out of these common differentially methylated CpGs we selected those that fulfilled two criteria: 1) there were at least three consecutive CpGs probes with a significant difference, 2) the median methylation level of all of the three subgroups was either lower or higher than that of the controls (filter 2). Consecutive CpGs were defined as occurring next to each other based on their genomic coordinates; they may not be representative of true consecutive CpGs in the genome, as those CpGs not included in the array as well as those discarded in the quality control steps were not considered. The filtering process is shown in Fig. 1.

The output list of significant CpGs that passed the filtering process was subjected to further selection by comparing the median methylation level of each of the subgroups to controls (Supplementary Table 1). We set a threshold of 5% methylation difference to further narrow down significant probes. The *HOXA4* differentially methylated area was defined by the CpGs that passed the filtering process, comprising altogether 12 + 3 consecutive CpG sites.

### DNA methylation analysis of *HOXA4* region with EpiTYPER

We measured the methylation level of CpGs in the *HOXA4* differentially methylated area using Sequenom EpiTYPER (Sequenom, San Diego, CA, USA). The analysis was carried out on 44 SRS patients, one individual with UPD(7)pat, 39 SGR patients, and 16 controls. Bisulfite conversion with the EZ-96 Methylation Kit (Zymo research corporation), subsequent sample processing, and quality control were carried out at the Karolinska Institutet Mutation Analysis Core Facility. All assays were performed in technical duplicates, and mean of the duplicate measurements was used in the analyses. If one of the duplicate samples failed, then data from the one informative sample only was used. One control sample, one SGR sample and the duplicate of one 11p15 LOM SRS sample were excluded due to experimental failure. Normality of the data was tested using Shapiro-Wilk test of normality and homogeneity of variances by Levene’s statistic test. Data were further analyzed by one-way ANOVA in SPSS. Dunnett t-tests, which compare all patient groups against the control group, were performed post hoc.

### Individual methylation level analysis and hypomethylated status

For each CpG in the *HOXA4* differentially methylated area, and each EpiTYPER site, standard deviation was calculated from the methylation level of the 16 control samples. Normal distribution of the control group data for each CpG (or a combination of CpG sites in Epityper site HOXA4_F6_CpG_12.13.14) was tested by Shapiro-Wilk normality test. Individual SD values for the SRS patients were calculated based on the standard deviation of the controls. A CpG site was considered hypomethylated when the methylation level was below -2 SD. We considered an individual patient generally hypomethylated at *HOXA4* when the methylation level was below -2 SD for at least two-thirds of the CpG sites. For the Illumina assay, the treshold was 10 out of 15 CpG sites (67%), and for the EpiTYPER assay 3 our of 4 sites (75%).

Similarly, the patients were tested for 11p15 LOM in preliminary stages of the data analysis. We considered an individual patient having 11p15 LOM when at least two-thirds or 24 out of 36 (67%) CpG sites were hypomethylated at chr11:2019090–2023450 in hg19 build.

### Correlation analysis of *HOXA4* methylation level and height in healthy children

Illumina 450 K methylation data from 227 healthy children from the BAMSE cohort were used to study methylation level of each of the *HOXA4* differentially methylated area CpGs in relation to their height measurements. Both height measurements and DNA samples for methylation analysis were obtained at approximately age 8. Additionally, records of birth length, height at 4 y and height at 16 y were obtained^43^. Height measurements were converted to units of standard deviation (SD) for exact age and gender to perform correlation analysis with methylation values. Pearson product-moment correlation coefficient was computed for CpGs with normally distributed data. Normality of the data was tested with Kolmogorov-Smirnov test of normality. Additional correlation analyses were performed by computing Spearman rank-order correlation coefficient for all CpGs in the *HOXA4* region of interest. The analysis was performed for each of the CpGs in comparison to birth length and height measurements at each age in SPSS.

### Correlation analysis of *HOXA4* expression and methylation level

Expression data was taken from our previous publication, where expression of *HOXA4* in blood was studied by qPCR^17^. The expression data from nine UPD(7)mat SRS patients, one individual with UPD(7)pat and ten controls were analyzed for correlation with each of the 16 *HOXA4* CpGs listed in Supplementary Table 5. Normality of the data was tested with Shapiro-Wilk test of normality. As expression data was not normally distributed, Spearman rank-order correlation analysis was performed. To calculate average methylation level of the 16 *HOXA4* CpGs for each individual, the methylation levels were first converted to SD scale, based on the standard deviation of the 10 controls.

### Data availability

The Illumina 450 K BeadChip methylation data discussed in this publication have been deposited in NCBI’s Gene Expression Omnibus^44^ and are accessible through GEO Series accession number GSE104451 (https://www.ncbi.nlm.nih.gov/geo/query/acc.cgi?acc=GSE104451).

## Electronic supplementary material

Supplementary information
