# Supplementary material for: Hypomethylation of HOXA4 promoter is common in Silver-Russell syndrome and growth restriction and associates with stature in healthy children
Source: Sci Rep. 2017 Nov 16;7:15693. doi: 10.1038/s41598-017-16070-5 (PMC5691194; doi:10.1038/s41598-017-16070-5)
Supplement: Supplementary file 1 — Supplementary information [file 41598_2017_16070_MOESM1_ESM.pdf]

Supplementary information for:

Hypomethylation of *HOXA4* promoter is common in Silver-Russell syndrome and growth restriction and associates with stature in healthy children

Mari Muurinen (1), Katariina Hannula-Jouppi (1, 2), Lovisa E. Reinius (3), Cilla Söderhäll (3, 4), Simon Kebede Merid (5), Anna Bergström (5, 7), Erik Melén (5, 6, 7), Göran Pershagen (5, 7), Marita Lipsanen-Nyman (8), Dario Greco (9,10), Juha Kere (1, 3, 11, *)

1 Folkhälsan Institute of Genetics, Helsinki, and Research Programs Unit, Molecular Neurology, University of Helsinki, Helsinki, Finland
2 Department of Dermatology, Skin and Allergy Hospital, University of Helsinki and Helsinki University Hospital, Helsinki, Finland
3 Department of Biosciences and Nutrition, Karolinska Institutet, Huddinge, Sweden
4 Department of Women's and Children's Health, Karolinska Institutet, Stockholm, Sweden
5 Institute of Environmental Medicine, Karolinska Institutet, Stockholm, Sweden
6 Sachs' Children's Hospital, Södersjukhuset, Stockholm, Sweden
7 Centre for Occupational and Environmental Medicine, Stockholm County Council, Stockholm, Sweden
8 Children's Hospital, University of Helsinki and Helsinki University Hospital, Helsinki, Finland
9 Institute of Biotechnology, University of Helsinki, Helsinki, Finland
10 Faculty of Medicine and Life Sciences & Institute of Biosciences and Medical Technology, University of Tampere, Finland
11 Department of Medical and Molecular Genetics, King's College London, Guy's Hospital, London, UK

*Corresponding author: juha.kere@ki.se

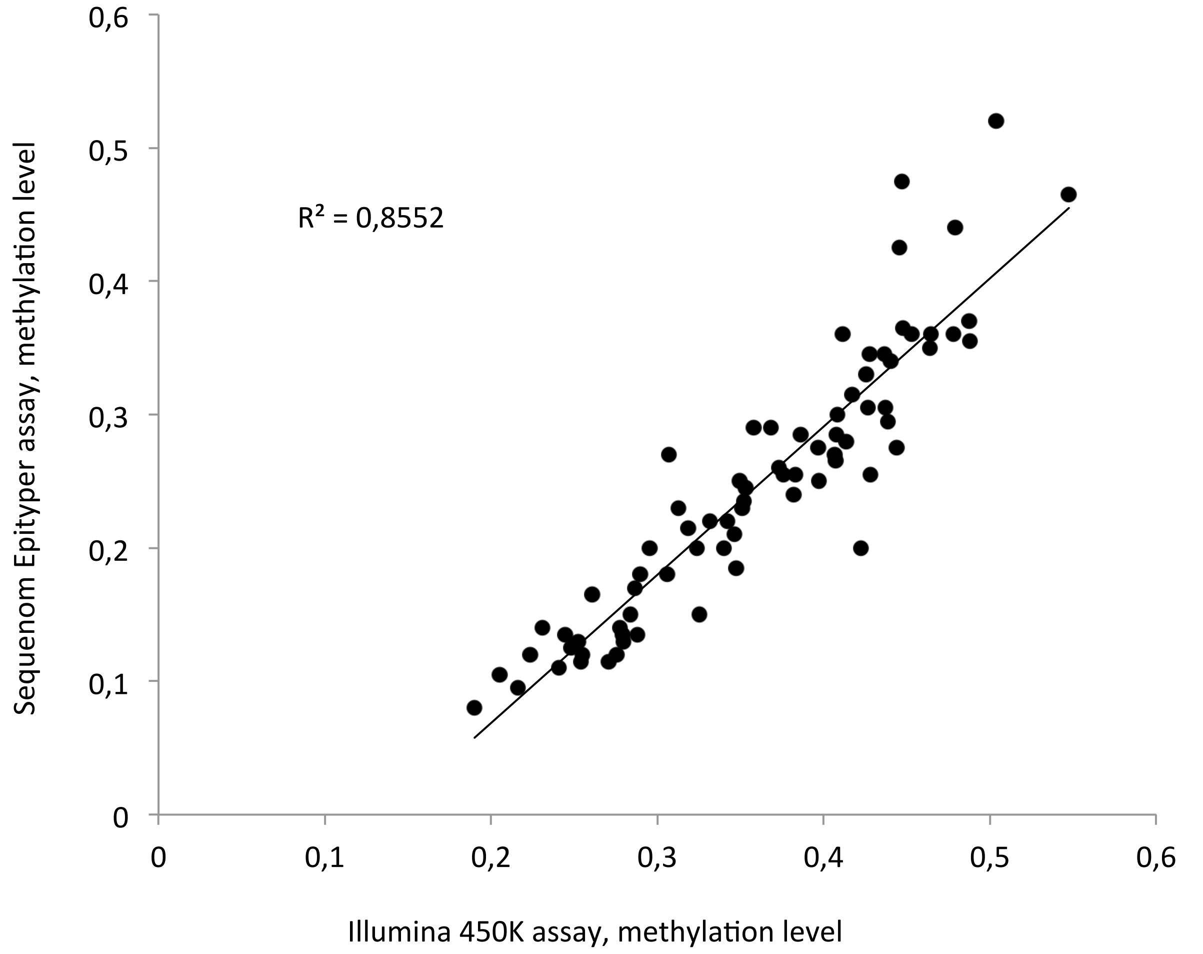

Supplementary figure 1. Correlation of methylation level measured by Epityper assay and Illumina 450K assay for the CpG at chr7:27170313 (cg04317399)

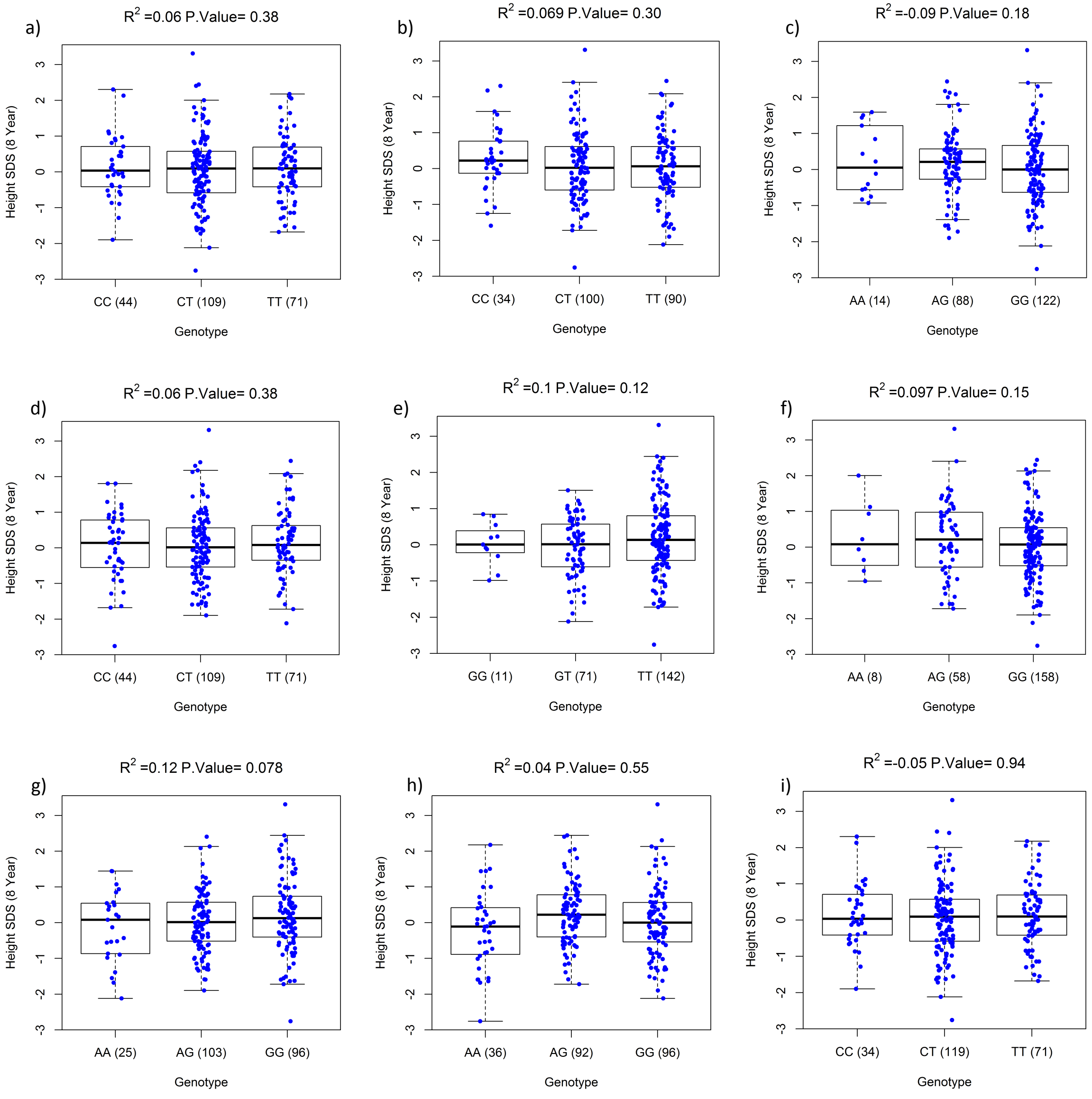

Supplementary figure 2. Association of height and genotype of height-associated SNPs in healthy controls of the Bamse cohort. Height has been measured approximately at the age of eight and measurements converted into SD scale. SNPs: a) rs1042725, b) rs2282978, c) rs2562784, d) rs8007661, e) rs11107116, f) rs2814993, g) rs10946808, h) rs6854783, i) rs6060369.

Supplementary table 1. Output list of all CpGs that passed the filtering process of 450K BeadChip assay. Median methylation values for controls (n=16), UPD(7)mat (n=10), clinical SRS (n=13) and 11p15 LOM (n=21) are represented. Methylation value for the single UPD(7)pat sample is also included. Difference in median methylation level (Δβ*100) between each group and the controls is higlighted in green when the difference <2% (absolute methylation percentage), and in red when the difference >5% (absolute methylation percentage). Horizontal dashed line represents the breakpoint where *HOXA4* filtered CpGs were not consecutive.

Gene	locus	Coordinates in hg19	CpG ID	controls	UPD(7)mat		clinical SRS		11p15 LOM		UPD(7)pat
				median β	median β	Δβ*100	median β	Δβ*100	median β	Δβ*100	β
AGL	1p21.2	100315729	cg08457409	0,02700422	0,03106522	0,406099547	0,02927068	0,226646053	0,03146936	0,446514449	0,0487343
AGL	1p21.2	100315927	cg04143346	0,03271391	0,03761182	0,489791077	0,03648421	0,377030014	0,03597946	0,326554695	0,04303187
AGL	1p21.2	100315971	cg17474694	0,017791	0,02593256	0,814156174	0,02296944	0,517844126	0,02302037	0,522937028	0,02714439
FAM98A	2p22.3	33824395	cg06227257	0,01771466	0,02885952	1,11448577	0,02455451	0,683984366	0,02539969	0,768503054	0,02475127
FAM98A	2p22.3	33824427	cg16512069	0,05212739	0,05622932	0,41019351	0,05514752	0,302013996	0,05531415	0,318676188	0,04959107
FAM98A	2p22.3	33824431	cg18977641	0,03873951	0,04578876	0,704924705	0,04303603	0,429651646	0,04284431	0,410479347	0,04908338
EXOC6B	2p13.2	72946664	cg23684316	0,59330972	0,55750627	-3,58034453	0,58288525	-1,04244679	0,56960811	-2,37016049	0,55992722
EXOC6B	2p13.2	72977657	cg03526652	0,91803561	0,90631738	-1,17182353	0,90013526	-1,79003514	0,90751185	-1,05237594	0,91748152
EXOC6B	2p13.2	72980252	cg22043275	0,9273877	0,92164293	-0,57447686	0,92327361	-0,41140906	0,92240129	-0,49864124	0,9123978
TMEM42/ MIR564	3p21.31	44903463	cg17410922	0,01971145	0,02815576	0,844431496	0,02505004	0,533859193	0,02553179	0,582034623	0,0261009
TMEM42	3p21.31	44903516	cg26889990	0,02627839	0,03757216	1,129377515	0,03373567	0,745727736	0,03086689	0,458849959	0,035739
TMEM42	3p21.31	44903551	cg24100506	0,1013754	0,11099755	0,962215668	0,11420343	1,282803364	0,11347572	1,210032349	0,11926623
intergenic	5p15.33	3180947	cg19598479	0,92438129	0,89855519	-2,58260919	0,90890054	-1,54807449	0,89753832	-2,68429673	0,91132921
intergenic	5p15.33	3181925	cg18894214	0,64226355	0,55996695	-8,22965974	0,58301147	-5,92520825	0,57219683	-7,00667188	0,6411336
intergenic	5p15.33	3182057	cg06976144	0,57952205	0,50158956	-7,79324821	0,49293873	-8,65833134	0,51925021	-6,02718326	0,59174962
intergenic	5p15.33	3182108	cg10463207	0,76051471	0,66919105	-9,13236606	0,68784817	-7,26665457	0,71309048	-4,74242353	0,72550485
PCDHB3	5q35.1	140479025	cg23918315	0,31395218	0,40365934	8,970716216	0,38509867	7,114649217	0,36043194	4,647976341	0,62204091
PCDHB3	5q35.1	140479979	cg18782991	0,08790681	0,09820896	1,030214724	0,09291149	0,500468355	0,09710328	0,919647451	0,16306068
PCDHB3	5q35.1	140479983	cg02125910	0,12913986	0,15138665	2,224679094	0,14790102	1,876116301	0,15060559	2,146573321	0,25499015
ZNF354C	5q35.3	178487310	cg19761115	0,02433059	0,02913152	0,480092445	0,02634467	0,201407265	0,02708241	0,275181945	0,02929912
ZNF354C	5q35.3	178487410	cg25942031	0,01935395	0,02437628	0,502232899	0,02130004	0,19460897	0,02471592	0,536196796	0,03184542
ZNF354C	5q35.3	178487576	cg07515940	0,04982951	0,05355741	0,372789713	0,05807547	0,824595732	0,06043144	1,060192742	0,05170251
intergenic	6p22.1	28584121	cg20839206	0,33964339	0,36303928	2,339589002	0,39059383	5,095043579	0,37282714	3,318374914	0,44304833
intergenic	6p22.1	28584138	cg22121557	0,16388157	0,17231315	0,843158342	0,237245	7,336342501	0,16597589	0,209431906	0,20682112
intergenic	6p22.1	28584155	cg03858673	0,14656134	0,15847624	1,191490072	0,19155112	4,498977374	0,17217122	2,560987871	0,18470743
TOMM6	6p21.1	41755749	cg06805345	0,03828057	0,04081941	0,253883744	0,04051181	0,223123358	0,04183282	0,355224758	0,04879169
TOMM6	6p21.1	41757330	cg06487442	0,87839034	0,86138106	-1,70092783	0,87454587	-0,38444665	0,87083634	-0,75539995	0,88410739
(next to TOMM6)	6p21.1	41758316	cg26004268	0,92833912	0,91291216	-1,5426955	0,92737383	-0,09652865	0,92586752	-0,24716037	0,93577286
KHDRBS2	6q11.1	62995963	cg18239753	0,10024395	0,09404723	-0,61967202	0,09141659	-0,88273591	0,09342632	-0,68176331	0,13889235
KHDRBS2	6q11.1	62996022	cg16587616	0,08533196	0,08772047	0,238850942	0,08883311	0,350114442	0,09303705	0,770509308	0,16981207
KHDRBS2	6q11.1	62996119	cg26715952	0,05843981	0,05173929	-0,67005246	0,04771021	-1,07296012	0,05352747	-0,49123403	0,08408501
C7orf20	7p22.3	921092	cg23397909	0,95388827	0,93560699	-1,82812814	0,9448417	-0,90465718	0,94260342	-1,12848538	0,9459073
C7orf20	7p22.3	921156	cg20776543	0,95633259	0,9394422	-1,68903979	0,94709727	-0,92353225	0,94183049	-1,45021047	0,95601521
C7orf20	7p22.3	921845	cg23237765	0,77211962	0,83081168	5,86920547	0,84227113	7,015150606	0,82109765	4,897803276	0,85329493
HOXA4	7p15.2	27169674	cg11532431	0,85141185	0,68984574	-16,1566104	0,76030761	-9,11042356	0,78642891	-6,49829377	0,89300425
HOXA4	7p15.2	27169740	cg00562553	0,8549225	0,76764232	-8,72801714	0,78344087	-7,14816265	0,80474686	-5,01756405	0,87549968
HOXA4	7p15.2	27170241	cg22997113	0,75003674	0,56856631	-18,1470436	0,60784311	-14,2193629	0,65535283	-9,46839097	0,76124925
HOXA4	7p15.2	27170313	cg04317399	0,42394877	0,28166768	-14,2281088	0,3272419	-9,6706868	0,36205022	-6,18985481	0,42813642
HOXA4	7p15.2	27170388	cg07317062	0,5004445	0,30263461	-19,7809895	0,3592233	-14,1221208	0,41879428	-8,16502236	0,51614853
HOXA4	7p15.2	27170394	cg19142026	0,42784812	0,18110267	-24,6745442	0,22116936	-20,6678756	0,32266609	-10,5182024	0,44274331
HOXA4	7p15.2	27170412	cg11410718	0,55407327	0,33689131	-21,7181957	0,38788151	-16,6191757	0,44517356	-10,8899715	0,5462584
HOXA4	7p15.2	27170717	cg17457637	0,41249307	0,26520791	-14,7285163	0,30916349	-10,3329578	0,35393141	-5,85616566	0,43508919
HOXA4	7p15.2	27170819	cg06942814	0,53752563	0,42205883	-11,5466807	0,44535832	-9,21673111	0,48162604	-5,58995929	0,54667345
HOXA4	7p15.2	27170832	cg08657492	0,63450817	0,43518972	-19,9318451	0,4788654	-15,5642764	0,54581548	-8,8692686	0,6240739
HOXA4	7p15.2	27170880	cg04321618	0,44215924	0,31093317	-13,1226071	0,3450797	-9,70795408	0,37759473	-6,45645113	0,46086227
HOXA4	7p15.2	27170892	cg14359292	0,37096889	0,19475379	-17,6215094	0,22751582	-14,3453072	0,29860079	-7,23680964	0,41349459
HOXA4	7p15.2	27170994	cg24169822	0,5003774	0,30192753	-19,8449873	0,34403913	-15,6338278	0,39930922	-10,1068184	0,52615346
HOXA4	7p15.2	27171051	cg17591595	0,76872748	0,66828177	-10,0445709	0,6533831	-11,5344387	0,70811706	-6,06104218	0,78592302
HOXA4	7p15.2	27171154	cg11908057	0,84586862	0,77774883	-6,81197922	0,76932173	-7,65468977	0,80298622	-4,28824047	0,87928123
SEPT7	7p14.2	35840513	cg17692200	0,03714071	0,04442006	0,727934835	0,04494641	0,780569796	0,04635414	0,92134277	0,02659296
SEPT7	7p14.2	35840534	cg07462932	0,02062471	0,02678955	0,616483926	0,03077139	1,014668204	0,02709278	0,646807016	0,03773847
SEPT7	7p14.2	35840614	cg21458073	0,02030712	0,02489844	0,459132071	0,02922594	0,89188188	0,02622465	0,591752562	0,03420399
SYPL1	7q22.3	105752648	cg14114033	0,0465794	0,05321224	0,663283853	0,05753601	1,095661213	0,05383595	0,725654945	0,05778413
SYPL1	7q22.3	105752920	cg08159831	0,05124604	0,06073169	0,948564881	0,06096873	0,972269257	0,05839567	0,71496279	0,05356808
SYPL1	7q22.3	105752950	cg24140629	0,01766709	0,02170785	0,40407632	0,03203745	1,437035955	0,02623039	0,85633006	0,03259988
CCAR1	10q21.3	70481015	cg09933375	0,03102002	0,04221601	1,11959896	0,04093666	0,991664225	0,03858716	0,756714303	0,05396052
CCAR1	10q21.3	70481056	cg14623805	0,04802089	0,05500859	0,698770598	0,05602317	0,800227761	0,05528601	0,726512626	0,04910672
CCAR1	10q21.3	70481448	cg15947099	0,04920143	0,05718848	0,798705351	0,06704029	1,783886538	0,06297723	1,377580433	0,06478024
SBF2	11p15.4	10315754	cg04415045	0,02646204	0,03604813	0,958608255	0,02799783	0,153578351	0,03248118	0,601914177	0,0335297
SBF2	11p15.4	10315759	cg04313601	0,01707315	0,02127807	0,420491968	0,02055443	0,348127297	0,02071264	0,363948777	0,02467506
SBF2	11p15.4	10315761	cg26975351	0,0214235	0,03105183	0,962833865	0,02447033	0,304683763	0,02706036	0,563686733	0,04003213
SBF2	11p15.4	10315914	cg01222603	0,01903755	0,02408961	0,505205421	0,0252705	0,623294348	0,02271478	0,367722048	0,02779186
HOXC8	12q13.13	54403178	cg02344911	0,05811968	0,05999831	0,187862644	0,06122353	0,310384996	0,07054091	1,242122834	0,09199748
HOXC8	12q13.13	54403254	cg05022306	0,03691178	0,04086109	0,394931057	0,04027167	0,335988301	0,03975679	0,284500509	0,04577613
HOXC8	12q13.13	54403314	cg19634247	0,0793595	0,08202438	0,266487762	0,08175922	0,239971322	0,08250429	0,314478166	0,09444207
EEA1	12q22	93323267	cg18580865	0,05746306	0,06424879	0,678573033	0,06511097	0,764790977	0,06191641	0,445334809	0,05765298

EEA1	12q22	93323273	cg00140235	0,05989214	0,07723201	1,7339872	0,06697895	0,708680938	0,07492424	1,503210683	0,06891763
EEA1	12q22	93323276	cg11643463	0,01686483	0,02166734	0,480250338	0,01928046	0,241562725	0,02305902	0,619418746	0,02013048
TMEM62	15q15.2	43425591	cg02257533	0,04380553	0,04857827	0,477273886	0,04906932	0,526379389	0,04807121	0,42656823	0,05698485
TMEM62	15q15.2	43425644	cg11184154	0,028106	0,03816653	1,006052949	0,03443497	0,63289722	0,03651831	0,841231776	0,03734723
TMEM62	15q15.2	43425698	cg21541406	0,03051882	0,0385164	0,799757929	0,04247226	1,195344242	0,03279762	0,227879951	0,04213404
DUOX1/DUOXA1	15q21.1	45422054	cg24505618	0,09837758	0,10115284	0,27752613	0,0985795	0,020191958	0,10482055	0,64429686	0,11738599
DUOX1/DUOXA1	15q21.1	45422062	cg05804220	0,05187628	0,05379345	0,191717287	0,058052	0,617571614	0,0588128	0,693652247	0,06546444
DUOX1/DUOXA1	15q21.1	45422083	cg10496082	0,02333679	0,02706994	0,37331562	0,02945029	0,611349951	0,02999788	0,666108889	0,02986442
ALOX12B	17p13.1	7982773	cg05704893	0,05210596	0,05953752	0,743155466	0,05393622	0,183025715	0,05649147	0,438551068	0,06057261
ALOX12B	17p13.1	7982806	cg27619163	0,04355639	0,05403348	1,047709057	0,05381978	1,026339054	0,05291865	0,936225194	0,05099039
ALOX12B	17p13.1	7982898	cg25874034	0,05599857	0,06249253	0,649396391	0,0596896	0,369102675	0,06165944	0,566087575	0,0514447
CCR10/CNTNAP1	17q21.2	40834836	cg24213196	0,06002245	0,06372768	0,370523178	0,06477816	0,475571134	0,06660413	0,6581684	0,07597042
CCR10/CNTNAP1	17q21.2	40834984	cg03107659	0,04566444	0,04590329	0,023885481	0,05103244	0,536800456	0,05302699	0,736255043	0,06278209
CCR10/CNTNAP1	17q21.2	40835003	cg13052543	0,02575399	0,03023847	0,448448753	0,0300722	0,431821022	0,03240161	0,664762149	0,04583628
RPRML	17q21.32	45056634	cg16179976	0,02484074	0,03020001	0,535927057	0,02820822	0,336748451	0,02770668	0,286594091	0,0304294
RPRML	17q21.32	45056646	cg05955013	0,03223454	0,04226755	1,003300879	0,03916534	0,693080018	0,04181226	0,957772177	0,04235782
RPRML	17q21.32	45056795	cg05302784	0,03667496	0,04069536	0,402040388	0,0399717	0,329673737	0,04127702	0,460205919	0,04205833
SNRPD1	18q11.2	19192308	cg12351856	0,08393942	0,09372076	0,97813383	0,10528998	2,135056221	0,09026154	0,632211995	0,0926109
SNRPD1	18q11.2	19192384	cg17797182	0,05831564	0,06644402	0,812838848	0,06107692	0,276128856	0,06272329	0,440765541	0,05760342
SNRPD1	18q11.2	19192502	cg21774115	0,03305989	0,03957597	0,651608298	0,03819727	0,513738145	0,03808708	0,502719304	0,03839672
NXT1	20p11.21	23331282	cg26752655	0,05778631	0,06361233	0,582601608	0,06251635	0,473003358	0,06324033	0,545401406	0,06528438
NXT1	20p11.21	23331308	cg00982357	0,04205213	0,049318	0,726587642	0,05006575	0,801362243	0,05121845	0,916632952	0,05214387
NXT1	20p11.21	23331342	cg23040205	0,03107059	0,03872674	0,765614781	0,03997682	0,890622433	0,0389554	0,788480753	0,04798031
COL9A3	20q13.33	61447982	cg13900920	0,03802974	0,03864856	0,061881879	0,04595747	0,792772193	0,04522656	0,719681267	0,0469943
COL9A3	20q13.33	61447992	cg11698944	0,0293184	0,03372083	0,44024306	0,03137075	0,205234451	0,03537724	0,605883655	0,04155202
COL9A3	20q13.33	61448079	cg21686379	0,03956502	0,04279475	0,322972466	0,04068906	0,112403938	0,04446128	0,489625654	0,05231246

Supplementary table 2. Methylation status for each individual and *H19* gene region measured by Illumina 450K BeadChip assay. Hypomethylated sites, with methylation below -2SD of the control level, are marked in green. Hypermethylated sites, with methylation level above +2SD of the control level, are marked in red.

group	cg13210239	cg17985533	cg09701145	cg25437674	cg10154633	cg26469586	cg24409677	cg16303279	cg02694715	cg11735853	cg01977486	cg25838645	cg13581483	cg04088212	cg01539474	cg18362496	cg24605090	cg04817190	cg06749854	cg24510613	cg16675558	cg03996735	cg18104242	cg27300742	cg25281616	cg01895612	cg23476401	cg00237904	cg06765785	cg25821896	cg25579157	cg02886509	cg02657360	cg16574793	cg09452478	cg02864690
11p15 LOM																																				
11p15 LOM																																				
11p15 LOM																																				
11p15 LOM																																				
11p15 LOM																																				
11p15 LOM																																				
11p15 LOM																																				
11p15 LOM																																				
11p15 LOM																																				
11p15 LOM																																				
11p15 LOM																																				
11p15 LOM																																				
11p15 LOM																																				
11p15 LOM																																				
11p15 LOM																																				
11p15 LOM																																				
11p15 LOM																																				
11p15 LOM																																				
11p15 LOM																																				
11p15 LOM																																				
11p15 LOM																																				
clinical SRS																																				
clinical SRS																																				
clinical SRS																																				
clinical SRS																																				
clinical SRS																																				
clinical SRS																																				
clinical SRS																																				
clinical SRS																																				
clinical SRS																																				
clinical SRS																																				
clinical SRS																																				
clinical SRS																																				
clinical SRS																																				
UPD(7)mat																																				
UPD(7)mat																																				
UPD(7)mat																																				
UPD(7)mat																																				
UPD(7)mat																																				
UPD(7)mat																																				
UPD(7)mat																																				
UPD(7)mat																																				
UPD(7)mat																																				
UPD(7q31-qter)mat																																				
UPD(7)pat																																				
control																																				
control																																				
control																																				
control																																				
control																																				
control																																				
control																																				
control																																				
control																																				
control																																				
control																																				
control																																				
control																																				
control																																				
control																																				
control																																				

Supplementary table 3. Methylation status for each individual and *HOXA4* Epityper site. Hypomethylated sites, with methylation below -2SD of the control level, are marked in green. Hypermethylated sites, with methylation level above +2SD of the control level, are marked in red.

group	HOXA4_F6_CpG_1	HOXA4_F6_CpG_12.13.14	HOXA4_F6_CpG_15	HOXA4_F6_CpG_16
11p15 LOM				
11p15 LOM				
11p15 LOM				
11p15 LOM				
11p15 LOM				
11p15 LOM				
11p15 LOM				
11p15 LOM				
11p15 LOM				
11p15 LOM				
11p15 LOM				
11p15 LOM				
11p15 LOM				
11p15 LOM				
11p15 LOM				
11p15 LOM				
11p15 LOM				
11p15 LOM				
11p15 LOM				
11p15 LOM				
11p15 LOM				
clinical SRS				
clinical SRS				
clinical SRS				
clinical SRS				
clinical SRS				
clinical SRS				
clinical SRS				
clinical SRS				
clinical SRS				
clinical SRS				
clinical SRS				
clinical SRS				
clinical SRS				
UPD(7)mat				
UPD(7)mat				
UPD(7)mat				
UPD(7)mat				
UPD(7)mat				
UPD(7)mat				
UPD(7)mat				

UPD(7)mat				
UPD(7)mat				
UPD(7q31-qter)mat				
UPD(7)pat				
SGR				
SGR				
SGR				
SGR				
SGR				
SGR				
SGR				
SGR				
SGR				
SGR				
SGR				
SGR				
SGR				
SGR				
SGR				
SGR				
SGR				
SGR				
SGR				
SGR				
SGR				
SGR				
SGR				
SGR				
SGR				
SGR				
SGR				
SGR				
SGR				
SGR				
SGR				
SGR		NA		
SGR				
SGR				
SGR				
SGR	NA	NA	NA	NA
SGR				
SGR				
SGR				
control				
control				
control				
control				
control				
control				
control				
control				
control				
control	NA	NA	NA	NA
control				
control				
control				
control				
control				
control				

Supplementary table 4. List of height-associated SNPs.

SNP	Publications
rs1042725	Lettre G, Jackson AU, Gieger C, et al. Identification of ten loci associated with height highlights new biological pathways in human growth. Nature genetics. 2008;40(5):584-591. doi:10.1038/ng.125.
	Weedon MN, Lango H, Lindgren CM, et al. Genome-wide association analysis identifies 20 loci that influence adult height. Nature genetics. 2008;40(5):575-583. doi:10.1038/ng.121.
rs10946808	Lettre G, Jackson AU, Gieger C, et al. Identification of ten loci associated with height highlights new biological pathways in human growth. Nature genetics. 2008;40(5):584-591. doi:10.1038/ng.125.
rs6060369	Lettre G, Jackson AU, Gieger C, et al. Identification of ten loci associated with height highlights new biological pathways in human growth. Nature genetics. 2008;40(5):584-591. doi:10.1038/ng.125.
rs8007661	Lettre G, Jackson AU, Gieger C, et al. Identification of ten loci associated with height highlights new biological pathways in human growth. Nature genetics. 2008;40(5):584-591. doi:10.1038/ng.125.
rs314277	Lettre G, Jackson AU, Gieger C, et al. Identification of ten loci associated with height highlights new biological pathways in human growth. Nature genetics. 2008;40(5):584-591. doi:10.1038/ng.125.
rs2562784	Lettre G, Jackson AU, Gieger C, et al. Identification of ten loci associated with height highlights new biological pathways in human growth. Nature genetics. 2008;40(5):584-591. doi:10.1038/ng.125.
rs2282978	Weedon MN, Lango H, Lindgren CM, et al. Genome-wide association analysis identifies 20 loci that influence adult height. Nature genetics. 2008;40(5):575-583. doi:10.1038/ng.121.
rs2814993	Weedon MN, Lango H, Lindgren CM, et al. Genome-wide association analysis identifies 20 loci that influence adult height. Nature genetics. 2008;40(5):575-583. doi:10.1038/ng.121.
rs11107116	Weedon MN, Lango H, Lindgren CM, et al. Genome-wide association analysis identifies 20 loci that influence adult height. Nature genetics. 2008;40(5):575-583. doi:10.1038/ng.121.
rs6854783	Weedon MN, Lango H, Lindgren CM, et al. Genome-wide association analysis identifies 20 loci that influence adult height. Nature genetics. 2008;40(5):575-583. doi:10.1038/ng.121.

Correlation of methylation and expression level in *HOXA4*			
	**r**	**n**	**p**
cg11532431	-0,77	20	7,20E-05
cg00562553	-0,76	20	9,80E-05
cg22997113	-0,81	20	1,40E-05
cg04317399	-0,72	20	3,71E-04
cg07317062	-0,67	20	1,21E-03
cg19142026	-0,72	20	3,87E-04
cg11410718	-0,68	20	1,01E-03
cg17457637	-0,76	20	8,80E-05
cg06942814	-0,72	20	3,00E-04
cg08657492	-0,72	20	3,00E-04
cg04321618	-0,72	20	3,00E-04
cg14359292	-0,72	20	3,13E-04
cg25952581	-0,71	20	4,94E-04
cg24169822	-0,69	20	6,75E-04
cg17591595	-0,70	20	6,25E-04
cg11908057	-0,58	20	6,75E-03
Average methylation In SD scale	-0,75	20	1,38E-04

Supplementary table 5. Spearman correlation analysis for methylation level of *HOXA4* area CpGs and expression level of *HOXA4* in UPD(7)mat, UPD(7)pat and control samples.

Supplementary table 6. Clinical information on SRS patients.

Patient	Birth weight SD	Birth length SD	Birth weight and/or birth length less than or equal to -2SDS for gestational age	postnatal growth in SD at closest measurement to age 24 months	Postnatal growth less than or equal to -2SD in closest available measurement to age 24 months	leg and/or body asymmetry	protruding forehead	relative macrocephaly	feeding difficulties	Score (fulfilled criteria, out of 6)
11p15 LOM	-4,7	-6,9	+	-6,0	+	?	+	+	n/a	4
11p15 LOM	-3,2	-3,5	+	-2,4	+	+	+	+	-	5
11p15 LOM	-5,5	-6,4	+	-5,8	+	+	+	+	-	5
11p15 LOM	-1,8	-2,0	+	-3,2	+	+	+	+	-	5
11p15 LOM	-4,2	-6,7	+	-6,3	+	+	+	+	-	5
11p15 LOM	-4,8	-6,6	+	-5,8	+	+	+	+	+	6
11p15 LOM	-4,7	-7,2	+	-3,4	+	+	+	+	-	5
11p15 LOM	-3,8	-4,2	+	-5,0	+	+	+	+	+	6
11p15 LOM	-4,2	-5,8	+	-5,8	+	+	+	+	-	5
11p15 LOM	-4,6	-5,2	+	-3,7	+	+	+	+	+	6
11p15 LOM	-3,7	-3,3	+	-2,6	+	+	+	-	n/a	4
11p15 LOM	-5,2	-5,3	+	-4,8	+	+	+	n/a	-	4
11p15 LOM	-3,8	-4,3	+	-4,8	+	+	+	+	+	6
11p15 LOM	-4,0	-5,2	+	-4,2	+	+	+	+	+	6
11p15 LOM	-2,1	-1,7	+	-3,5	+	+	+	+	-	5
11p15 LOM	-3,6	-3,2	+	-4,2	+	-	+	+	+	5
11p15 LOM	-3,6	-4,9	+	-5,6	+	+	+	+	n/a	5
11p15 LOM	n/a	-4,0	+	-3,6	+	n/a	+	n/a	n/a	3
11p15 LOM	-5,0	-6,6	+	-4,0	+	+	+	+	-	5
11p15 LOM	-4,5	-4,7	+	-3,4	+	n/a	+	+	n/a	4
11p15 LOM	-3,3	-5,4	+	-4	+	+	+	+	n/a	5
clinical SRS	-4,3	-4,6	+	-3,6	+	+	+	+	n/a	5
clinical SRS	-3,6	-4,1	+	n/a	n/a	-	+	-	n/a	2
clinical SRS	-4,9	-4,6	+	-2,0	+	-	+	+	+	5
clinical SRS	-2,0	-2,3	+	-3,4	+	n/a	+	+	?	4
clinical SRS	-3,6	-4,1	+	-4,1	+	+	+	+	n/a	5
clinical SRS	-2,8	-2,4	+	-4	+	-	+	+	n/a	4
clinical SRS	-2,4	-2,5	+	-2,5	+	?	+	+	n/a	4
clinical SRS	-3,3	-4,7	+	-5,5	+	-	+	+	+	5
clinical SRS	-4,2	-5,7	+	-3,4	+	-	-	+	+	4
clinical SRS	-2,4	-3,4	+	-3,9	+	-	-	+	-	3
clinical SRS	-2,2	-1,2	+	-2,6	+	+	n/a	n/a	n/a	3
clinical SRS	-3,4	-4,5	+	-3,8	+	-	+	+	-	4
clinical SRS	-4,8	-5,8	+	-4	+	-	+	+	n/a	4
UPD(7)mat	-3,7	-4,8	+	-6,8	+	+	+	+	+	6
UPD(7)mat	-2,5	-3,8	+	-2,8	+	+	+	+	+	6
UPD(7)mat	-2,8	-1,8	+	-4,6	+	-	+	+	+	5
UPD(7)mat	-2,7	-3,4	+	-3,8	+	-	+	+	+	5
UPD(7)mat	-2,6	-2,0	+	-3,7	+	-	+	+	+	5
UPD(7)mat	-2,2	-3,5	+	-6,2	+	-	+	+	+	5
UPD(7)mat	-2,2	-3,2	+	-4,8	+	-	+	+	n/a	4
UPD(7)mat	-3,8	-4,1	+	-5,0	+	+	+	+	?	5
UPD(7)mat	-3,2	-3,7	+	-5,2	+	-	+	+	+	5
UPD(7q31-qter)mat	-4,5	-5,2	+	-3,0	+	-	+	+	+	5

Supplementary table 7. Clinical information on SGR patients.

Patient	Birth weight SD	Birth length SD	Birth weight and/or birth length less than or equal to -2SD for gestational age	postnatal growth in SD at closest measurement to age 24 months	Postnatal growth less than or equal to -2SD in closest available measurement to age 24 months
SGR	n/a	-2,9	+	-4,1	+
SGR	-4,9	-5,9	+	-3,8	+
SGR	-2,8	-2,9	+	-4,8	+
SGR	-2,8	-5,0	+	-4,7	+
SGR	-3,0	-4,6	+	-5,2	+
SGR	-2,6	-4,1	+	-3	+
SGR	-3,5	-4,0	+	-4,1	+
SGR	-3,3	-3,5	+	-4,5	+
SGR	-4,4	-6,3	+	-5,1	+
SGR	-3,8	-3,0	+	-2,7	+
SGR	-5,1	-6,4	+	-4,4	+
SGR	-2,7	-2,9	+	-4,0	+
SGR	-0,2	-1,3	-	-2,8	+
SGR	-1,2	-1,9	-	-2,4	+
SGR	-2,5	-2,9	+	-3,3	+
SGR	-1,1	-2,0	+	-3	+
SGR	-4,4	-4,9	+	-2,0	+
SGR	-1,3	-2,3	+	-2,9	+
SGR	-0,5	-1,7	-	-2,6	+
SGR	-1,1	-3,4	+	-3,4	+
SGR	-3,0	-4,8	+	-4,5	+
SGR	-0,3	-1,6	-	-4,2	+
SGR	-1,4	-1,1	-	-3,1	+
SGR	-2,2	-2,6	+	-2,9	+
SGR	-2,7	-2,1	+	-4,2	+
SGR	-0,6	0,6	-	-2,7	+
SGR	-4,0	-2,0	+	-3,6	+
SGR	-5,7	-6,9	+	-2,9	+
SGR	-1,8	-2,0	+	-3,0	+
SGR	-3,8	-3,7	+	-4,5	+
SGR	-2,2	-2,1	+	-5,4	+
SGR	-5,1	-6,6	+	-4,7	+
SGR	-2,9	-2,0	+	-4,9	+
SGR	-1,5	-1,4	-	-3,2	+
SGR	-2,3	-3,1	+	-2,8	+
SGR	-0,1	-1,2	-	-3,8	+
SGR	-4,1	-5,9	+	-5,5	+
SGR	-2,8	-4,9	+	-7,0	+
